# The Effect of Polydimethylsiloxane-Ethylcellulose Coating Blends on the Surface Characterization and Drug Release of Ciprofloxacin-Loaded Mesoporous Silica

**DOI:** 10.3390/polym11091450

**Published:** 2019-09-04

**Authors:** Adrianna Skwira, Adrian Szewczyk, Magdalena Prokopowicz

**Affiliations:** Department of Physical Chemistry, Faculty of Pharmacy, Medical University of Gdańsk, Hallera 107, Gdańsk 80-416, Poland

**Keywords:** coating, drug delivery, surface roughness, polymers, mesoporous silica

## Abstract

In this study, we obtained novel solid films composed of ciprofloxacin-loaded mesoporous silica materials (CIP-loaded MCM-41) and polymer coating blends. Polymer coating blends were composed of ethylcellulose (EC) with various levels of polydimethylsiloxane (PDMS, 0, 1, 2% (*v*/*v*)). The solid films were prepared via the solvent-evaporation molding method and characterized by using scanning electron microscopy (SEM), optical profilometry, and wettability analyses. The solid-state of CIP present in the solid films was studied using X-ray diffraction (XRD) and differential scanning calorimetry (DSC). The release profiles of CIP were examined as a function of PDMS content in solid films. The surface morphology analysis of solid films indicated the progressive increase in surface heterogeneity and roughness with increasing PDMS content. The contact angle study confirmed the hydrophobicity of all solid films and significant impact of both PDMS and CIP-loaded MCM-41 on surface wettability. DSC and XRD analysis confirmed the presence of amorphous/semi-crystalline CIP in solid films. The Fickian diffusion-controlled drug release was observed for the CIP-loaded MCM-41 coated with PDMS-free polymer blend, whereas zero-order drug release was noticed for the CIP-loaded MCM-41 coated with polymer blends enriched with PDMS. Both the release rate and initial burst of CIP decreased with increasing PDMS content.

## 1. Introduction

Local drug delivery systems have been widely applied in bone tissue diseases [1,2]. They require two most pressing goals to be achieved: a controlled initial release and long-term delivery of drug [3]. At present, mesoporous silica materials, e.g., MCM-41, have been studied as local antibiotic/anticancer drug delivery systems for the bone treatment of osteomyelitis or osteosarcoma due to their desirable features such as large surface area (~800 m^2^/g), uniform pore size (~2–6 nm), modifiable surface properties [4,5]. Moreover, mesoporous silica materials have been found as a nontoxic to the surrounding bone tissue and biocompatible with the osteoblast cells [6]. Furthermore, Braun et al. reported that mesoporous silica materials may release small amounts of Si (~60 ppm) through dissolution in simulated body fluid [7]. It has been confirmed that even relatively low concentration of Si has a positive impact on bone homeostasis and density [8]. However, another characteristic feature of many drug-loaded mesoporous silica materials is the high amount of drug released in a burst stage [9]. This can lead to a high local concentration of drug which may be cytotoxic to surrounding tissue. The reduction of burst release can be achieved by using polymeric coatings. 

Controlled local drug release provides a suitable dosing of drug in a specific area in a prolonged manner. The use of bone drug delivery systems which provide controlled release is considered as an optimal and benign treatment characterized by increased effectiveness, extended duration of therapeutic effect, and reduced adverse effects, e.g., toxicity [10,11]. Nowadays, polymeric coatings have been emphasized as an interesting way to modify drug release from mesoporous silica matrices providing a physical barrier between the drug entrapped in the pores and the fluids [12]. Different polymers, such as poly(ethylene glycol) (PEG) [13], Eudragit RL and Eudragit S [14] were used for this purpose. Furthermore, blends of two types of polymers, which are known to be non-toxic and exhibit different physicochemical characteristics can be also used [15]. In the polymer coatings the release profiles of drug primarily depend on the polymer/polymer blend ratio. By simply modifying the polymer/polymer blend ratio, the properties of the obtained film can be effectively altered and thus broad ranges of drug release profiles can be provided [15,16].

Apart from the appropriate drug release, another requirement for mesoporous silica materials as local drug delivery systems to bone is their integration with the host tissue. Surface roughness has been considered as a crucial limiting factor for applied materials in the context of physical and biological compatibility with surrounding tissue [17]. In recent years, many materials intended for implantation with various surface structure were designed and examined for cellular response, activity, and bone formation [18,19]. It was proved for endosseous dental implants that a relatively rough surface improves implant stability, promotes tissue ingrowth, and enhances bone apposition [19,20,21]. Multiple methods of textural property modification are available [22,23]. However, two primary trends can be distinguished: control of the porosity of a material during its synthesis (1), and surface post-treatment of a synthesized material (2) among which a coating has been considered as one of the most effective methods [24]. 

The main features of appropriate implantable coating mixtures are biocompatibility, biostability and desired mechanical properties [25]. These characteristics are well documented for polydimethylsiloxane (PDMS) [26] and ethylcellulose (EC) [27]. EC is the most stable cellulose derivative and a water-insoluble polymer that has been used in many pharmaceutical applications as a coating or time-release agent. In addition, EC, due to its function as binder, flexible film former, water barrier and rheology modifier, has been increasingly applied in bone tissue engineering [27,28,29]. PDMS is characterized by a water-insoluble hydrophobic nature, good adhesive and plasticizing capacity, which characterize its influence on drug release and the physical properties of coated materials [30].

Therefore, the use of PDMS:EC combinations as new coating blends for ciprofloxacin-loaded MCM-41 (CIP-loaded MCM-41) was investigated in this paper. As the MCM-41 has been previously well described as a drug delivery system, we decided to modify the drug release rate by using a polymeric coating. Ciprofloxacin was chosen as the model drug due to its broad spectrum activity and common application in osteomyelitis [31]. The main idea of our studies was to prepare novel solid films of polymer-coated CIP-loaded MCM-41 which would provide the desired prolonged drug release with low burst release in vitro. Furthermore, the physicochemical properties of the obtained solid films were thoroughly investigated. 

## 2. Materials and Methods 

### 2.1. Materials

Tetraethyl orthosilicate (TEOS), cetyltrimethylammonium bromide (CTAB), ethanol, aqueous ammonia (25%), hydroxyl-terminated polydimethylsiloxane fluid (PDMS, 65 cSt, d = 0.97 mg/mL) were all obtained from Sigma-Aldrich. Ethylcellulose (EC, Ethocel^®^ 20 cP) was obtained from Dow Chemical. Acidic solution of ciprofloxacin lactate (pH = 3.5, 10 mg/mL, Proxacin) was obtained from Polfa S.A. (Warsaw, Poland).

### 2.2. Synthesis of Mesoporous Silica Materials (MCM-41)

The synthesis of mesoporous silica materials, MCM-41, was carried out by a templating method using tetraethyl orthosilicate (TEOS) as a silica source and the cationic surfactant cetyltrimethyl- ammonium bromide (CTAB) as a template [32]. Briefly, 125 g of water, 12.5 g of ethanol, 9.18 g of ammonia aq. (25 %), and 2.39 g of CTAB were stirred together in polypropylene beaker on a magnetic mixer (300 rpm) at 25 °C for approx. 15 min until a homogenous solution was formed. The pH of the solution was 10. Then, 10.03 g of TEOS was added and the resulting mixture was continuously stirred for 2 h. Next, hydrothermal treatment of the mixture was carried out at 90 °C for 5 days without stirring. The resulting solid product was recovered by vacuum filtration, washed with 100 mL of absolute ethanol and dried at 40 °C for 1 h. The CTAB template was removed from the product by calcination in air at 550 °C (with heating rate of 1 °C/min) for a period of 6 h in a muffle furnace (FCF 7SM, CZYLOK, Jastrzebie-Zdroj, Poland). The final MCM-41 samples in powders form with the fraction size range of 200–500 µm were obtained by micronisation (Mortar Grinder Pulverisette 2, Fritsch, Weimar, Germany). The micronisation lasted 10 min at 50 rpm.

### 2.3. Ciprofloxacin Adsorption onto MCM-41

Ciprofloxacin (CIP) adsorption onto MCM-41 was performed according to the following procedure (the optimal conditions of adsorption process were determined in preliminary studies). Briefly, the synthesized MCM-41 (particles fraction in the range of 200 µm–500 µm) was immersed in acidic solution (pH = 3.5) of ciprofloxacin lactate (10 mg/mL) at ratio 100 mg of silica per 2 mL of solution and incubated with stirring (300 rpm) at 25 ± 0.5 °C for 3 h to ensure the equilibrium state. Then, CIP-loaded MCM-41 was centrifuged, separated from supernatant, and freeze-dried (−52 °C, 0.1 mBar, 24 h). The absorbance of the CIP remaining in the supernatant was measured spectrophotometrically at 278 nm (model UV-1800 UV-Vis spectrophotometer, Shimadzu, Kyoto, Japan). The amount of CIP adsorbed onto MCM-41 and adsorption efficiency were calculated using Equations (1) and (2), respectively:

(1) $$Q_{e}=\frac{(C_{0}-C_{e})\cdot V}{m}$$

(2) $$\%\mathrm{Ads}=\left(\frac{C_{0}-C_{e}}{C_{e}}\right)\cdot 100\%$$

where *Q*_e_ (mg/g) is an amount of CIP adsorbed at the equilibrium state, %Ads (%) is an adsorption efficiency coefficient, *C*_0_ (mg/L) is an initial CIP concentration, *C*_e_ (mg/L) is a CIP concentration at equilibrium state, *V* (L) is a volume of CIP solution, and m (g) is the mass of MCM-41 used.

### 2.4. Fabrication of Solid Films Composed of CIP-Loaded MCM-41 and Polymer Blends

The polymer coating blends were prepared by mixing 5% (*w*/*w*) ethylcellulose (EC) ethanolic solutions with 0, 1, 2% (*v*/*v*) of hydroxyl-terminated polydimethylsiloxane (PDMS, 65 cSt, *d* = 0.97 mg/mL), giving the various PDMS:EC ratios (Table 1). 95% (*v*/*v*) ethanol was used as a solvent in each formulation. The prepared coating blends were homogenized by sonication for 20 min using a cooling bath. The pure solid films of each formulation were prepared via solvent-evaporation molding method as follows. Equal volumes (250 µL) of F1, F2, and F3 coating blends (Table 1) were poured into polypropylene molds, and incubated till complete ethanol evaporation (30 ± 0.5 °C, 60 ± 10% relative humidity, 24 h). The end of the solidification of the polymer coating blends is regarded as the moment when no changes in weight (within the limits of instrumental error (± 0.01 g)) were detected. After complete evaporation of the ethanol, the PDMS:EC ratios in the pure solid films changed from 1:99 and 2:98 (*v*/*v*, before solvent evaporation) into 1:4 and 1:2 (*w*/*w*, after solvent evaporation) for F2 and F3, respectively (Table 1).

A constant amount of CIP-loaded MCM-41 (200 µm–500 µm) was immersed in each polymer blend at a ratio 6 mg per 250 µL. Solid films of CIP-loaded MCM-41 coated with F1, F2, and F3 (MCM-F1, MCM-F2, MCM-F3) were fabricated in the same manner as pure solid films. The obtained solid films of F1, F2, and F3 and MCM-F1, MCM-F2, MCM-F3 were then removed from the molds and stored in desiccators at room temperature for further analyses.

### 2.5. Physicochemical Characterization

Pure solid films of F1, F2, and F3 were characterized using the Fourier transform infrared spectroscopy (FTIR, Jasco FT/IR-4200, Jasco, Pfungstadt, Germany). Samples for analysis were prepared using the KBr tablet technique. For a better comparison, the spectra of F2 and F3 were normalized to a maximum absorption of the dominant peak at 804 cm^−1^, attributed to the Si-C stretching in the Si–CH_3_ group [33].

The morphological characterization of MCM-F1, MCM-F2, and MCM-F3 solid films was carried out by using scanning electron microscopy (SEM). For comparative purposes, SEM analysis of CIP-loaded MCM-41 (before polymer coating) and pure solid films of F1, F2, F3 was performed. In order to identify the components of pure solid films, the samples were analyzed using the energy-dispersive X-ray spectroscopy (EDX). The samples for SEM were fixed on carbon tape and sputter coated with gold, prior to the analysis. SEM images was obtained on electron microscope (Hitachi SU-70, Japan) at an acceleration voltage of 3 kV.

The images of surface roughness with quantitative data of MCM-F1, MCM-F2, MCM-F3 solid films were obtained using an optical profilometer (Contour GTK, Bruker, Billerica, MA, USA). To obtain quantitative characterization, mapping of 46.9 µm × 62.5 µm size surface was done for 10 samples of each formulation and the average values together with standard deviations of roughness parameters were calculated. 

Wettability studies of solid films of F1, F2, F3, and MCM-F1, MCM-F2, MCM-F3 were performed by contact angle measurement at room temperature (DSA G10 goniometer, Kruss GmbH, Hamburg, Germany). The images of sessile drop of water and diiodomethane at the point of intersection (three-phase contact points) between the drop contour and the surface (baseline) were recorded for drop shape analysis (DSA) and determination of contact angle. The contact angle value was determined as an average of 10 measurements of each sample. 

The X-ray diffraction (XRD) analysis of F3, MCM-F3, CIP-loaded MCM-41, and CIP (as reverence) and differential scanning calorimetry (DSC) of F3, MCM-F3, CIP-loaded MCM-41, CIP, and MCM-41 (as references) were performed to characterize solid-state of CIP present in the obtained solid films. XRD data were collected using diffractometer (Empyrean PANalytical, Malvern, UK) with a CuKα radiation beam operating at 40 kV and 40 mA, in the 2*θ* range between 5° and 70° with a step width of 0.02° and at a scanning rate of 0.5°/min. DSC measurements were carried out using a DSC instrument (822e Mettler Toledo, Columbus, Ohio, USA). Samples of about 5 mg were placed in an aluminum pan with a hole in the lid. The experiments were performed under an N_2_ atmosphere (20 mL/min gas flow rate). The thermal behavior of the samples was investigated by heating the samples from 25 to 230 °C with 10 °C/min step. Calibration of the instrument was performed using an indium standard. 

### 2.6. Drug Release Analysis

The release study was performed under “sink” conditions (considering approx. 100 mg/mL solubility of CIP in water [34]) according to the following procedure. Each solid film of MCM-F1, MCM-F2, MCM-F3 was immersed in 2 mL of purified water and shaken at 37 ± 0. 5°C (80 rpm) for 7 days. The release medium was exchanged every 24 h, and the amount of CIP released was measured spectrophotometrically at wavelength of 278 nm (Shimadzu model UV-1800 UV-Vis spectrophotometer). Quantitative determinations of the amount of released CIP were based on pre-calibration of the spectrometer using standard water solutions of the CIP. 

The results were obtained from data groups of n = 7 and expressed as mean values ± standard deviation. The release kinetic parameters were calculated using linearized forms of Korsmeyer-Peppas, Higuchi models and zero order kinetics presented in Equations (3)–(5), respectively:

(3) logQ=logk+nlogt

(4) $$Q=k_{H}t$$

(5) $$Q=Q_{0}+k_{0}t$$

where *Q* (%) denotes the fraction released over time t (h; days), *Q*_0_ (%) is the initial fraction of released drug, n is a release exponent related to the drug release mechanism, *k* (h^−n^; days^−n^) is a rate constant, the *k*_H_ is a Higuchi dissolution constant (h^−1/2^; days^−1/2^), and k_0_ is the zero order release constant (% of dose released per day). In Equation (3), n < 0.5 indicates quasi-Fickian diffusion, *n* = 0.5 indicates a Fickian diffusion, for n between 0.5 and 1, the drug release is considered as non-Fickian diffusion, and *n* = 1 corresponds to zero-order release of case II diffusion. To find out the mechanism of drug release, the data for first 60% of drug release fraction (*Q*) were fitted with Korsmeyer-Peppas and Higuchi models.

## 3. Results and Discussion

### 3.1. Ciprofloxacin Adsorption onto MCM-41

The CIP adsorption onto successfully synthesized MCM-41 was verified by the Fourier transform infrared spectroscopy technique (Jasco model 4700 FTIR). The characteristic bands of the CIP molecule were observed what confirmed the presence of drug onto MCM-41 after the loading procedure (Supplementary Materials). The adsorption efficiency was 56 ± 2% what corresponded to the mean amount of CIP-loaded of 112 ± 4 mg per each 1 g of MCM-41.

### 3.2. Solid Films Formation

The pure solid films of three formulations of polymer blends were obtained via solvent-evaporation molding method (F1, F2, F3). The solid films of CIP-loaded MCM-41 coated with polymer blends (MCM-F1, MCM-F2, MCM-F3) were obtained in the same manner. The final thickness and mass of the pure solid films of F1, F2, F3 were ranged from 30 to 40 µm, and 10.0 to 14.6 mg, respectively, whereas solid films of MCM-F1, MCM-F2, MCM-F3 were characterized by thicknesses ranging from 50 to 60 µm and masses ranging from 16.0 to 20.6 mg.

### 3.3. Physicochemical Characterization

#### 3.3.1. FTIR Characterization

**Figure 1:**
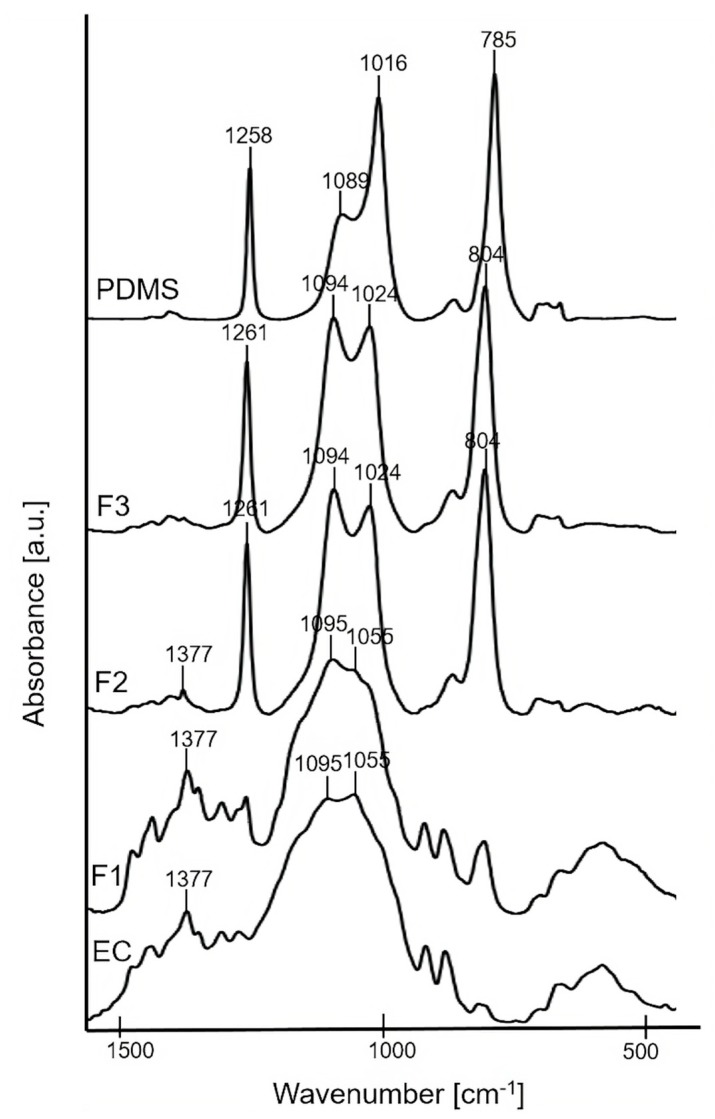
FTIR spectra of F1, F2, F3 solid films, and precursors: EC and PDMS.

The bulk molecular structure of F1, F2, and F3 pure polymer solid films was characterized using FTIR technique (Figure 1). FTIR spectra of the precursors (EC powder and PDMS liquid) are presented for comparative purposes. The spectrum of F1 (the formulation containing 100% of EC) showed the characteristic broad peaks at ~1055 cm^−1^ and ~1095 cm^−1^ attributed to C–O–C stretching vibrations, and ~1377 cm^−1^ attributed to C–H bending vibrations in the EC molecule [35]. For the pure solid films of F2, F3, the characteristic peak at ~1024 cm^−1^ corresponding to Si–O–Si asymmetrical stretching [36] confirmed the presence of PDMS. In addition, sharp peaks at ~1261 cm^−1^ attributed to Si–C stretching and ~804 cm^−1^ attributed to CH_3_ deformation in Si–CH_3_ group of PDMS were noticed [33]. The intensity of the peak at ~1024 cm^−1^ slightly increased for F3 compared to F2, thus confirming a higher content of PDMS in the F3 sample. Additionally, a small peak at ~1377 cm^−1^ of EC is observed for F2, what confirmed the higher content of EC in F2 compared to F3. For both F2 and F3, a peak at ~1094 cm^−1^ was also observed due to the overlapping of Si–O–Si asymmetrical stretching at ~1089 cm^−1^ from the PDMS together with the C–O–C stretching mode at ~1095 cm^−1^ from the EC.

#### 3.3.2. Surface Morphology

**Figure 2:**
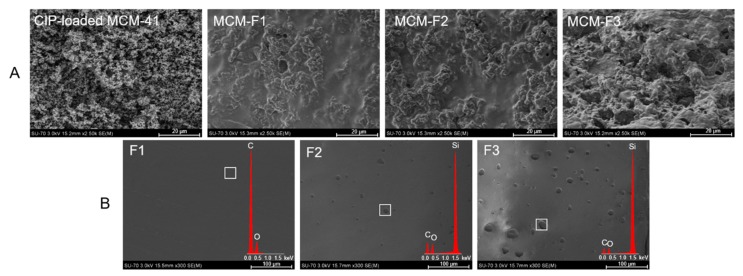
SEM images of CIP-loaded MCM-41, and solid films of: MCM-F1, MCM-F2, MCM-F3 (**A**); F1, F2, F3 (**B**).

The morphology of MCM-F1, MCM-F2, MCM-F3 solid films was characterized using SEM analysis (Figure 2). The SEM image of CIP-loaded MCM-41 powder before coating is also presented for comparative purposes (Figure 2A). The CIP-loaded MCM-41 powder was characterized by spherical particles loosely connected to each other. After coating with polymer blends the structure of the obtained solid films was more compact and heterogeneous. Moreover, the results suggested that the surface roughness increases with the increase of PDMS content in the solid films (Figure 2A). In order to confirm the observed differences in surface roughness the SEM-EDX analysis of pure solid films of F1, F2, F3 was also performed (Figure 2B). As it can be seen, the film of EC without PDMS (F1) was characterized by a smooth, gapless surface, whereas F2 and F3 present plain surface structure with an increasing number of cavities distributed in the solid films. The higher the PDMS content in polymer blend, the greater the number of cavities that was observed, indicating that they could be attributed to PDMS droplets (also confirmed by EDX profile in which the presence of silicon in F2 and F3 was noticed). It should be noted that no cavities attributed to PDMS were observed for MCM-F2, MCM-F3 solid films (Figure 2A) contrary to pure solid films of F2, F3 (Figure 2B). This may be related to occlusion of PDMS in the MCM-41 particles.

**Figure 3:**
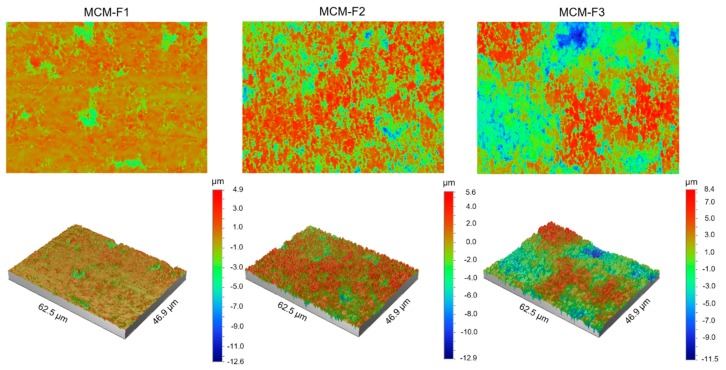
The optical profilometer images of 62.5 µm × 46.9 µm surfaces of MCM-F1, MCM-F2, MCM-F3 solid films.

The quantitative characterization of the surface of MCM-F1, MCM-F2, MCM-F3 solid films was performed by using optical profilometry. Table 2 shows the roughness parameters (*R*_a_—average roughness, *R*_q_—root-mean-square roughness, and *R*_t_—peak-valley difference) calculated over the whole tested area (46.9 µm × 62.5 µm) as the means of 10 samples of each formulation together with standard deviations. As presented in Table 2, the quantitative results confirmed the increase of surface roughness in the following order: MCM-F1 < MCM-F2 < MCM-F3. The *R*_a_ value increased with the increase of PDMS content in solid film from 0.57 ± 0.10 µm for MCM-F1 to 2.49 ± 0.54 µm for MCM-F3. The highest values of roughness parameters were observed for MCM-F3 and correlated with the biggest cavities (Figure 3). These data indicate that the surface roughness of solid films is significantly dependent on the PDMS content in polymer blend what might be a promising feature in the context of tissue ingrowth. According to numerous in vitro experiments, implant roughness may affect osteoblast morphology, growth, proliferation and differentiation. It has been highlighted that the cells cultured on the rough surfaces show better morphology and proliferation in comparison to the controls cultured on flat surfaces [37].

#### 3.3.3. Contact Angle and Wetting Properties

In order to verify the wettability of the solid films of F1, F2, F3, and MCM-F1, MCM-F2, MCM-F3 a contact angle analysis was performed. The average contact angles and surface free energies determined for all samples are shown in Table 3. All of the examined samples were characterized as hydrophobic. For the pure solid films of F1, F2, F3 (Section A in Table 3) the most hydrophilic surface was observed for F1. As expected, for F2 the addition of hydrophobic hydroxyl-terminated PDMS resulted in a higher water contact angle value, suggesting an increase in hydrophobicity, compared to F1. Conversely, for F3 a slight decrease of water contact angle and increase of the polar component of the surface free energy were noticed, compared to F2. This can be explained by gaining insight into the molecular structure of hydroxyl-terminated PDMS which contains hydrophobic PDMS-rich domains (polydimethylsiloxane chains) terminated with hydrophilic hydroxyl groups. For MCM-F1, MCM-F2, MCM-F3 solid films (Section B in Table 3) the water contact angle increased, compared to pure solid films, due to the presence of CIP-loaded MCM-41. Relatively the most hydrophobic surface was found for the solid film of MCM-F1 (without PDMS). The increase of polar component of surface energy as a function of PDMS content was observed. This can be explained by the high adhesion of polydimethylsiloxane chains to hydrophobic CIP-loaded MCM-41. This behaviour results in an increase of the number of hydrophilic hydroxyl groups of PDMS oriented to the sample surfaces and thus an increase of surface polar components.

#### 3.3.4. Solid-State Characterization of Ciprofloxacin

**Figure 4:**
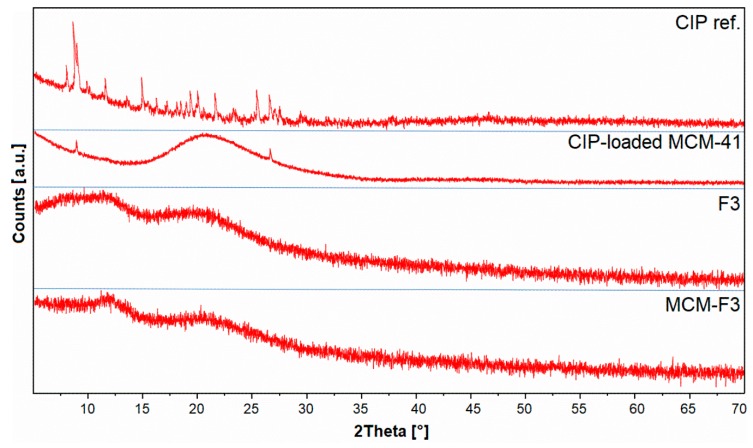
The XRD patterns of solid films of MCM-F3 and F3, CIP-loaded MCM-41, and CIP reference.

The XRD patterns of solid films of MCM-F3 and F3 (as representative examples), CIP-loaded MCM-41 and freeze-dried CIP reference are presented in Figure 4. The freeze-dried CIP reference was characterized by numerous well-defined sharp diffraction peaks demonstrating the crystalline nature of the drug. For the CIP-loaded MCM-41 sample the broad halo in the range of 15-35° derived from the amorphous silica with significant reduction of peaks characteristic for the CIP. Two diffraction peaks at 8 and 27° 2*θ* observed in CIP-loaded MCM-41 sample suggested the semi-crystalline nature of CIP molecules adsorbed onto the silica surface [38]. In the case of both the F3 and MCM-F3 the XRD patterns showed two broad amorphous halos at 10-15° and 15-30° 2*θ* which clearly revealed the amorphous nature of the obtained films.

**Figure 5:**
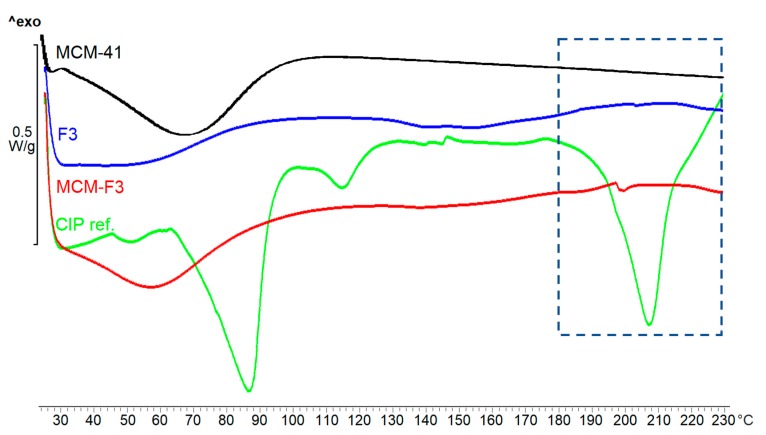
The DSC thermograms of solid films of MCM-F3, F3, unloaded MCM-41 and CIP reference.

The DSC thermograms of solid films of MCM-F3, F3, unloaded MCM-41, freeze-dried CIP (as references) are presented in Figure 5. As selected in the frame the freeze-dried CIP was characterized by the endothermic peak of melting point at approx. 207 °C. MCM-F3 exhibited a small endothermic peak shifted to lower temperatures (approx. 199 °C) corresponding to the melting of drug, which suggested the presence of the CIP in the amorphous or semi-crystalline state what was also confirmed by the XRD results (Figure 4). The amorphous nature of drug loaded into the mesoporous silica is already known phenomenon [39]. Moreover, the obtained DSC results are in accordance with the observations of Mesallati et al. [34] who have claimed that solid dispersions of CIP with various polymers displayed a clear melting endotherm of the drug, which could be taken as confirmation of the amorphous nature of the CIP inside the polymer matrices.

### 3.4. Ciprofloxacin Release

**Figure 6:**
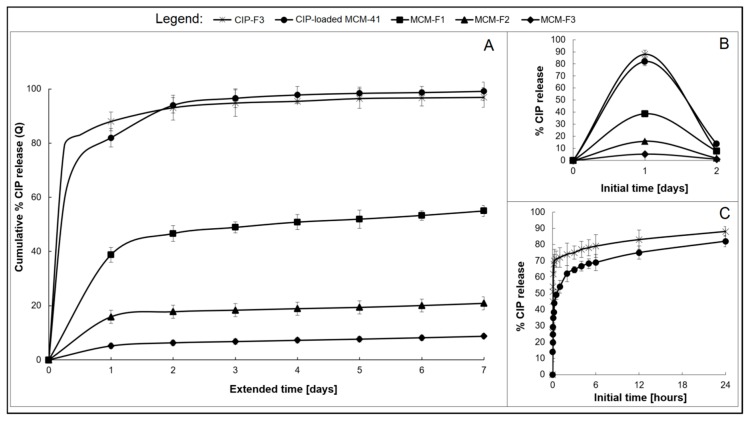
The overall CIP release profiles from MCM-F1, MCM-F2, MCM-F3, CIP-F3 solid films, and CIP-loaded MCM-41 (**A**); Insets: not-cumulative CIP release during the initial time: 0–2 days; (**B**) and initial 24 h CIP release from CIP-loaded MCM-41 and CIP-F3 (**C**).

The drug release was studied for MCM-F1, MCM-F2, MCM-F3 solid films to verify the impact of PDMS content on release profiles. Each solid film contained 6 mg of CIP-loaded MCM-41, corresponding to 0.67 ± 0.1 mg of CIP. CIP-elution profiles describing the cumulative percent amount of CIP released as a function of time (1–7 days), are presented in Figure 6A. Inset B shows the non-cumulative percent amount of released CIP at the sampling point as a function of the initial time period (0–2 days, Figure 6B). The CIP release from the CIP-loaded MCM-41 (6 mg, 200 µm–500 µm; in the powder form) and from solid film F3 with addition of pure CIP (CIP-F3, 0.67 ± 0.2 mg of CIP) are shown in Figure 6 for comparative purposes. As presented in Figure 6C, both CIP-loaded MCM-41 and CIP-F3 solid film were characterised by a relatively rapid release of CIP with a huge burst during the first 24 h (80 and 88%, respectively) with almost complete CIP release after 7 days (98 and 95%, respectively). In contrast, the initial amount of the drug eluted during the first day (initial burst) was reduced to 39% of the total amount of loaded CIP for the MCM-F1 solid film (PDMS-free, Figure 6B). Next, the release kinetics slowed-down and after four exchanges of the medium, the mean value of the recovered CIP from MCM-F1 corresponded to 50% of the total amount of loaded CIP (Figure 6A), whereas it was 75% after 39 daily exchanges (estimated from the release profile). In the case of the PDMS-containing solid films F2 and F3, the initial burst was reduced to 16% and 5% of the total amount of loaded CIP (corresponding to 107.5 and 33.6 µg of CIP released during the first day), respectively (Figure 6B). Then the release kinetics gently slowed down (Figure 6A). The *t*_25%_, *t*_50%_, and *t*_75%_ values (corresponding to 25%, 50% and 75% of the total amount of loaded CIP, respectively), calculated from the release profiles, were 15, 66, and 107 days or 46, 117, and 168 days for MCM-F2 and MCM-F3 solid films, respectively. Thus, the release of CIP from MCM-F1, MCM-F2, MCM-F3 solid films presented a significant modification compared to CIP-loaded MCM-41 and CIP-F3 solid film. The initial burst of CIP was reduced with the increase in content of PDMS in solid films. That is why the greatest reduction in initial burst was reported for MCM-F3 solid film characterized by a prolonged release rate similar to already exist antibiotic drug delivery systems [40].

The calculated kinetic parameters for the obtained release profiles are summarized in Table 4. It can be observed that all release profiles of solid films (excluding the first 24 h initial drug release) were characterized by zero order kinetics (*R*^2^ ≥ 0.89) with constant drug release rates per day. It should be noted that zero-order release CIP kinetics were observed for all PDMS-containing solid films, even after 7 days of release studies. Moreover, the release profiles of solid films were well described by both the Higuchi model (*R*^2^ ≥ 0.93) and the release exponent n values ≤ 0.55 suggesting the release of drug is controlled by diffusion of CIP from a non-disintegrating matrix [41]. However the use of CIP-loaded MCM-41 in solid films instead of pure CIP seemed to be essential in order to reduce the burst release, what was manifested in reduction of *k*_H_ values from 27.01 to 8.92 for CIP-F3 and MCM-F1 solid films, respectively.

Taking into account both the CIP release profile and the kinetic models the possible mechanism of drug release from CIP-loaded MCM-41 coated with polymer blends can be described as diffusion controlled [42]. In our case the water-soluble CIP molecules loaded into the mesoporous silica channels are entrapped in the EC-PDMS polymeric film which acts as a water-insoluble matrix. The structure of the formed EC film is dependent on the polymer-solvent interaction parameters. Jones et al. found that after ethanol evaporation, the structure of EC is heterogeneous, with numerous nanopores [43]. This confirms the release of CIP via simple diffusion from CIP-loaded MCM-41 coated with EC (MCM-F1 solid film) through the formed pores (release exponent *n* = 0.51, calculated from the Korsmeyer-Peppas model). However, the observed reduction of both the initial burst and the rate of CIP release from CIP-loaded MCM-41 coated with PDMS:EC blends may be attributed to the adhesion of PDMS chains onto the CIP-loaded MCM-41 surface, which is also in agreement with the obtained SEM results (Figure 2A) and wettability data (Table 3). Another reason can be related to a “locking” of the pore surface of EC due to the greater surface contact and thus easier penetration of the PDMS into the pores. The effect of PDMS on the release rate of water-soluble drugs from silica/PDMS composites has previously been reported [39]. It was found that an increase in PDMS content resulted in a decreasing rate of drug release, and as reported here, with a simultaneous increase in the values of the release exponent n. This phenomenon was correlated with a decrease in the porosity of the studied silica/PDMS composites, explained by the effect of the occlusion of hydrophobic PDMS-rich domains on the silica particles that may delay the penetration of water molecules into the composites, and hence dissolution of a loaded drug [44,45]. To sum up, the increase of PDMS content in solid films of MCM-F2, MCM-F3 may result in an increasing number of PDMS-filled nanopores, and also lock the CIP in the pores of MCM-41, thus slowing down the drug release [46]. Consequently, the zero-order CIP release could be explained by the very small magnitude of the interfacial partition coefficient of the drug and the small thickness of the drug depletion layer.

## 4. Conclusions

Novel solid films were obtained by coating CIP-loaded MCM-41 with PDMS:EC blends. Solid films with various levels of PDMS addition (0, 1, 2% (*v*/*v*)) were characterized in terms of their physicochemical properties. SEM and optical profilometry results indicated a progressive increase in the surface heterogeneity and roughness for the solid films of CIP-loaded MCM-41 coated with polymer blends as a function of PDMS content. The presence of amorphous/semi-crystalline CIP in the obtained films was confirmed by DSC and XRD analyses. Release studies showed a prolonged CIP release from all solid films for over 7 days with Fickian diffusion-controlled drug release for the EC formulation without PDMS and the zero-order drug release for formulations containing PDMS. This is probably related to the adhesion of hydrophobic chains of PDMS onto CIP-loaded MCM-41. The physicochemical characterization of the obtained solid films showed that by simple modification of the PDMS:EC ratio, the roughness and release profile of drug adsorbed into the MCM-41 pores can be easily altered. As the surface roughness has a fundamental significance for the integration with host bone tissue, the provided mesoporous silica coated with polymer blends could be a promising drug delivery system. The most beneficial physicochemical properties in the context of further biological evaluation, such as the greatest reduction of initial burst of CIP and the highest values of roughness parameters were observed for MCM-F3 solid film. The findings presented in this paper may be an excellent starting point for further investigations on the biocompatibility and material/cell interactions of the systems.

## Figures and Tables

**Table 1:** Composition of each formulation (F1–F3) with PDMS:EC ratio before (*v*/*v*) and after solvent evaporation (*w*/*w*).

**Before solvent evaporation**			
**Formulation**	**PDMS:EC ratio**	**PDMS content [µL]**	**EC ethanolic solution content [µL]**
F1	0:100	-	250.0
F2	1:99	2.5	247.5
F3	2:98	5.0	245.0
**After solvent evaporation**			
**Formulation**	**PDMS:EC ratio**	**PDMS content [mg]**	**EC content [mg]**
F1	0:1	-	10.0
F2	1:4	2.4	9.9
F3	1:2	4.8	9.8

**Table 2:** Mean roughness parameters with standard deviations of surface of MCM-F1, MCM-F2, MCM-F3 solid films.

Formulation	*R*_a_ ± SD [µm]	*R*_q_ ± SD [µm]	*R*_t_ ± SD [µm]
MCM-F1	0.57 ± 0.10	0.86 ± 0.17	11.22 ± 2.49
MCM-F2	1.86 ± 0.40	2.30 ± 0.45	19.12 ± 2.85
MCM-F3	2.49 ± 0.54	3.14 ± 0.70	23.67 ± 6.06

SD—standard deviation; *R*_a_—average roughness; *R*_q_—root-mean-square roughness; and *R*_t_—peak-valley difference.

**Table 3:** Average contact angle and surface free energy (*γ*_s_) with its dispersive (*γ*_s_^d^) and polar (*γ*_s_^p^) components of solid films of F1, F2, F3 (**A**), and MCM-F1, MCM-F2, MCM-F3 (**B**).

	Sample	Average Contact Angle [*θ*, °]		Surface Free Energy [mJ/m^2^]		
		Measuring Liquid				
		Water	Diiodomethane	Total (*γ*_s_)	Dispersive (*γ*_s_^d^)	Polar (*γ*_s_^p^)
A	F1	82.2	63.0	29.27	21.35	7.92
	F2	86.3	54.2	32.03	27.81	4.22
	F3	83.5	54.4	32.50	26.99	5.51
B	MCM-F1	112.9	81.9	16.40	16.07	0.33
	MCM-F2	125.1	67.5	29.28	27.97	1.31
	MCM-F3	124.5	62.9	32.86	31.21	1.65

**Table 4:** The kinetic parameters of fitted experimental data for MCM-F1, MCM-F2, MCM-F3, CIP-F3 solid films and CIP-loaded MCM-41.

Formulation	Korsmeyer-Peppas Model		Higuchi Model		Zero Order Kinetics *	
	*n*	*R* ^2^	*k*_H_	*R* ^2^	*k*_0_	*R* ^2^
CIP-F3	0.51	0.95	27.01	0.94	0.03	0.89
CIP-loaded MCM-41	0.34	0.92	4.93	0.85	0.02	0.82
MCM-F1	0.17	0.97	8.92	0.93	1.60	0.99
MCM-F2	0.33	0.87	6.80	0.98	0.61	0.99
MCM-F3	0.55	0.93	6.49	0.98	0.48	0.99

*R*^2^—coefficient of determination; *n*—release exponent in Korsmeyer-Peppas model; *k*_H_—Higuchi dissolution constant (h^−1/2^; days^−1/2^); *k*_0_—zero order release constant (% of dose released per day); * Calculated excluding the first 24 h initial release of CIP.

## Supplementary Materials

The following are available online at https://www.mdpi.com/2073-4360/11/9/1450/s1.
