# Supplementary figures and images for: The Effect of Polydimethylsiloxane-Ethylcellulose Coating Blends on the Surface Characterization and Drug Release of Ciprofloxacin-Loaded Mesoporous Silica
Source: Polymers (Basel). 2019 Sep 4;11(9):1450. doi: 10.3390/polym11091450 (PMC6780097; doi:10.3390/polym11091450)

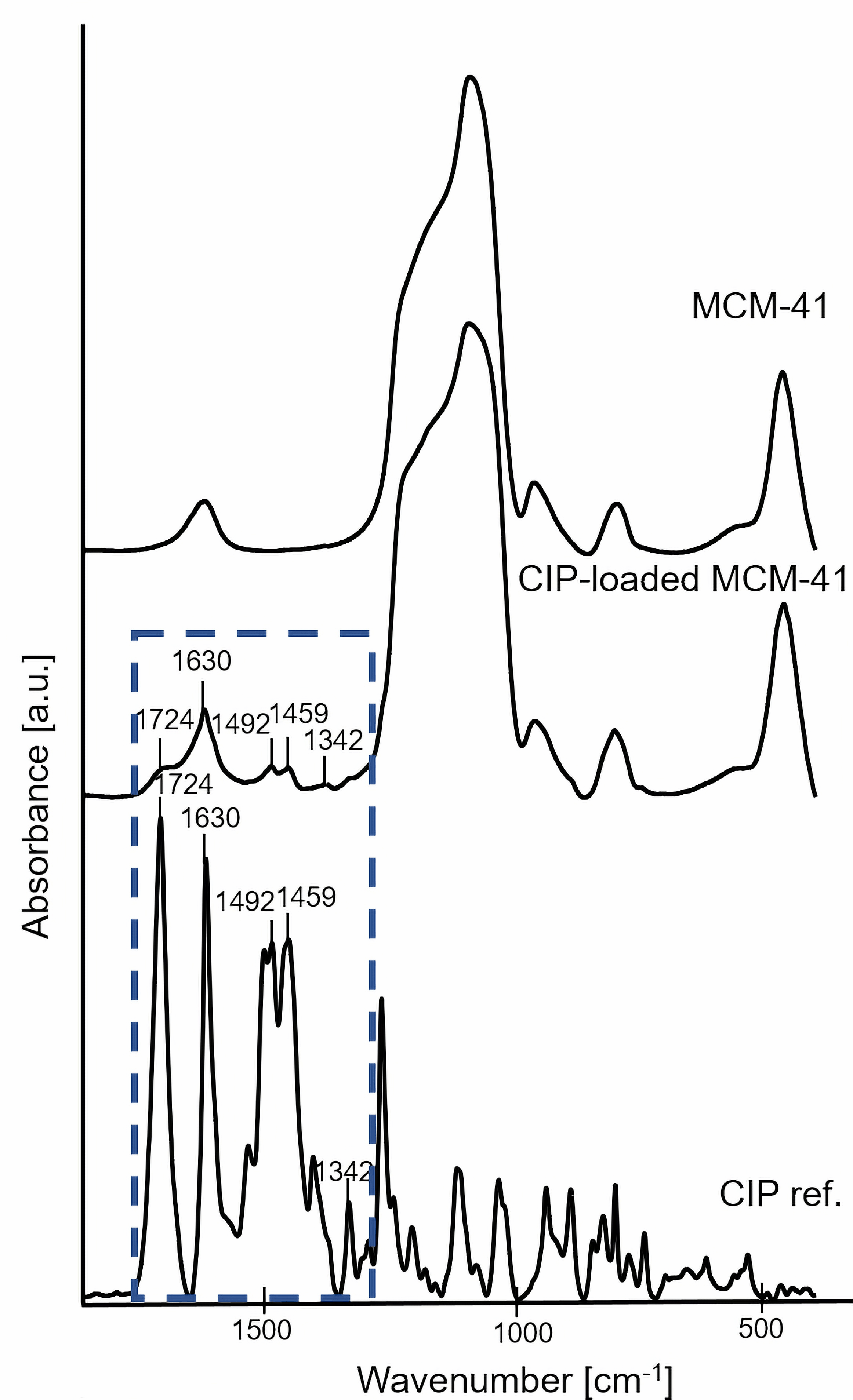

Supplement: Supplementary file 1 [file polymers-11-01450-s001.zip › polymers-582704-supplementary.gif]
